# Phlebosclerotic colitis: Our clinical experience of 25 patients in China

**DOI:** 10.1097/MD.0000000000012824

**Published:** 2018-10-26

**Authors:** Wenguo Chen, Huatuo Zhu, Hongtan Chen, Guodong Shan, Guoqiang Xu, Lihua Chen, Fei Dong

**Affiliations:** a Department of Gastroenterology, The First Affiliated Hospital; b Department of Radiology, The Second Affiliated Hospital, College of Medicine, Zhejiang University, Hangzhou, Zhejiang Province, China.

**Keywords:** calcification, computed tomography, herbal medicine, ischemic colitis, phlebosclerotic colitis

## Abstract

The aim of the current study was to enhance the awareness of phlebosclerotic colitis (PC) through our clinical experience.

A retrospective review of 25 patients who were diagnosed as PC in our 2 affiliated hospitals from January 2013 to October 2017 was conducted.

The patients were found at a mean age of 63.5 years, range 47 to 87years. The majority of patients were male (23 cases). Only 4 patients (16%, 4/25) had the history about long-term use of Chinese herbs and medical liquor. The most common symptoms were abdominal pain (40%) and intestinal obstruction (16%), followed by diarrhea (12%), and gastrointestinal bleeding (12%), etc. Three cases (12%) had no symptoms. The varying degrees of calcifications along the colon and mesenteric venous were found in all of their computed tomography (CT) images. The lesions mainly located in transverse and ascending colon (60%, 15/25). The terminal ileum, the whole colon and rectum involvement were also been found. Fourteen patients had the examination of colonoscopy which all presented characteristic dark purple-colored endoscopic findings. Conservative treatment with close follow-up was preferred in our group. Three cases had the surgery of colectomy due to the repeatedly intestinal obstruction, perforation.

The PC was a very rare but characteristic entity with unclear etiopathogenesis. Examination of abdomen CT and colonoscopy could help you to make clinical diagnosis.

## 1 Introduction

Phlebosclerotic colitis (PC), also known as mesenteric phlebosclerosis, is a unique form of ischemic colitis characterized by thread-like calcifications along affected colonic wall and mesenteric vein, dark-purple colored mucosa on colonoscopy, which involves the right hemicolon preferentially. Differentiated from the traditional ischemic colitis, its clinical presentations are usually gradual and chronic. This disease is considered to be irreversible, nonthrombotic, noninflammatory stenosis, or occlusion of the mesenteric veins.^[1]^ The etiology and pathogenesis of PC are not yet fully understood.^[2]^ To date, the majority of PC cases occurred in patients with Asian background, mostly from Japan.^[3]^ In China, more and more patients were diagnosed nowadays. However, characteristic computed tomography (CT) and endoscopic features may be missed or misdiagnosed due to the lack of knowledge of this rare entity.

Up to now, more and more literatures across the world described this entity, nevertheless the majority of articles were case reports. Herein, we retrospectively reviewed 25 cases of PC at our 2 affiliated hospitals over the last 5 years between 2013 and 2017. In our manuscript, we mainly analyzed their demographics, clinical manifestation, endoscopic and imaging characteristics, treatment, and discussed with reference to the literatures to enhance the awareness of the clinicians.

## 2 Materials and methods

A retrospective, descriptive study involving all patients with PC admitted to our 2 affiliated hospitals of Zhejiang University from January 2013 to October 2017 was conducted. A total of 25 patients were found at a mean age of 63.5 years, range 47 to 87years. The majority of patients were male (23 cases), only 2 females were found in our group. The symptoms included the following: abdominal pain in 10 cases, intestinal obstruction in 4 cases, chronic diarrhea in 3 cases, gastrointestinal bleeding in 3 cases, accidentally finding on CT scan in 3 cases, abdominal distension in 2 cases. The patients’ main characteristics are summarized in Table 1.

**Table 1:**
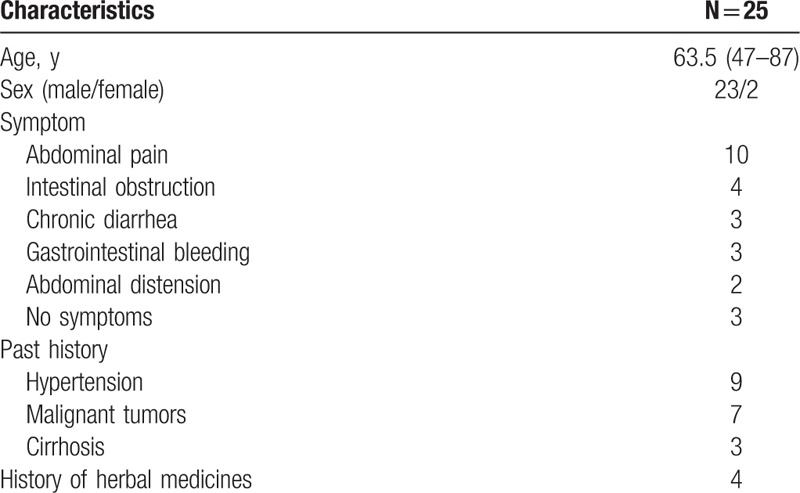
Patient characteristics.

We obtained human subjects approval from ethics committee of The First Affiliated Hospital, College of Medicine, Zhejiang University and The Second Affiliated Hospital, College of Medicine, Zhejiang University. The record/information of patients were anonymized and de-identified prior to analysis. Our institutional review board approved this study with a waiver of informed consent.

## 3 Results

### 3.1 General information

In our 25 cases, 9 patients had the history of hypertension, 7 patients with malignant tumors of digestive system, 3 patients with cirrhosis, etc. The tumors located in stomach (2 cases), pancreas (1 case), duodenum (1 case), rectum (1 case), and small intestine (1 case, stromal tumors). No patients found with definite history of thrombosis.

### 3.2 Radiographic results

**Figure 1:**
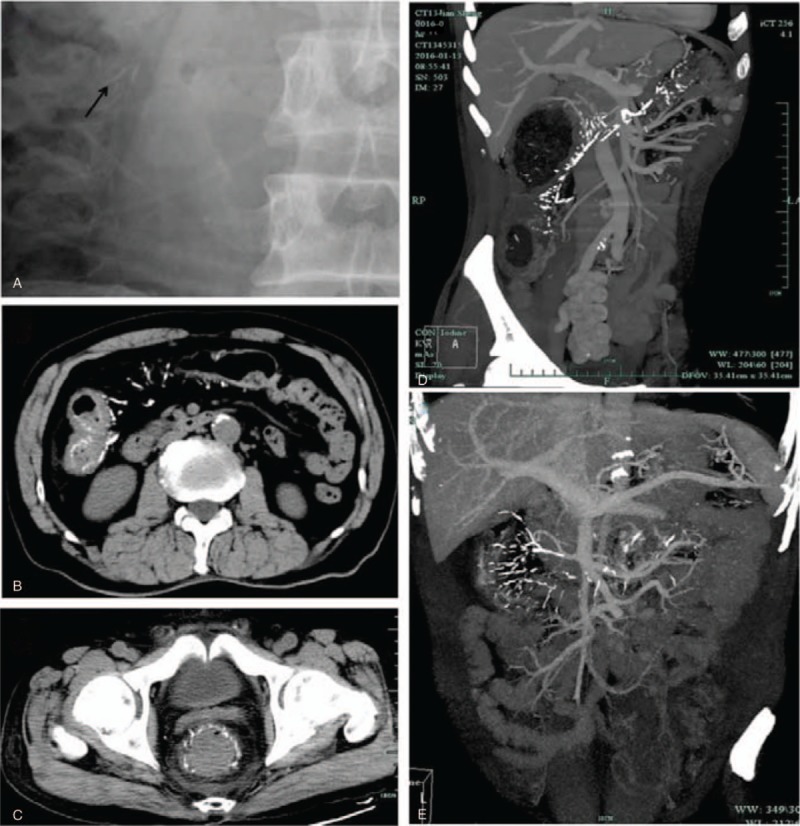
Characteristic radiologic findings. (A) Plain abdominal X-ray showed thread-like calcifications in the right hemicolon region (arrowheads). (B) On abdominal computed tomography (CT), wall-thickening and calcifications within the bowel wall and adjacent mesentery were observed. (C) A very rare phlebosclerotic colitis patient, linear calcifications extended into rectum. (D, E) Three-dimensional reconstruction of CT angiography depicted the precise extent of calcification.

Calcifications along the colon and marginal branches of mesenteric vein were its characteristic radiologic findings in common. Plain X-ray of the abdomen may showed multiple punctuate, thread-like or linear calcifications in the right hemicolon region which may easily be missed in the routine examination. Figure 1A showed the typical X-ray feature. Abdominal CT showed thickenings of colonic wall accompanied by mural and mesenteric venous calcifications (Fig. 1B, C). Increased density in the fatty tissue of the surrounding mesentery could be visualized also. The calcifications were more clearly seen on mesenteric side and primarily located perpendicular to the long axis of the colon. Further investigation such as computed tomography angiography (CTA) of mesenteric vessels could precisely identify the severity of the lesion and location of the calcifications involving intramural tributaries, marginal veins, and vena recti peripherally. Calcifications were found not only alongside the intramural and marginal mesenteric veins, but also extended into the superior (SMV) and inferior mesenteric veins (IMV) when it involved in more advanced phase affecting the entire colon. Figure 1 (D, E) depicted the 3-dimensional reconstruction image of CTA in patients with PC.

In our group, all the 25 patients received abdominal CT scan. The varying degrees of calcifications were encountered in each of them. Diffuse wall-thickening in the affected region were observed in 21 cases. The others showed calcifications with no obvious thickening of the colonic wall, of which 3 cases had no abdominal symptoms and 1 case had mild abdominal distension. Regarding the CT images, the calcification was not serious also in these 4 patients. The lesions mainly located in transverse and ascending colon (60%, 15/25). In 3 cases, lesions extended to the descending colon, and 3 cases of which involved the whole colon. In addition, terminal ileum, left hemicolon, and rectum involvement were also detected in our study.

### 3.3 Endoscopic view

**Figure 2:**
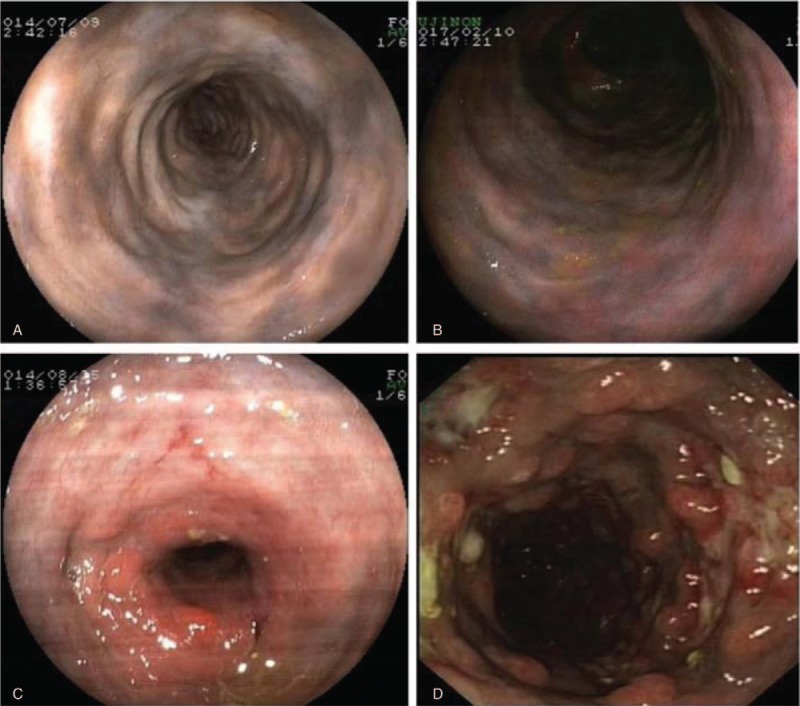
Characteristic endoscopic views. (A, B) Colonoscopy revealed bluish violet or dark-brownish mucosa in transverse colon (A) and ascending colon, ileocecal region (B). (C) Intestinal stenosis was noted in the hepatic flexure in patients with phlebosclerotic colitis (PC). (D) Colonoscopy showed dark purple appearance, with extensive erosions, ulcerations, and multi-focal nodular surface in a patient with PC.

A total of 14 patients had the examination of colonoscopy. Dark purple-colored mucosa was the most characteristic change, the color may be caused by chronic congestion with ischemia or by toxins that stained the bowel mucosa. This typical finding was presented on all our colonoscopy (100%, 14/14). Additionally, mucosal edema, erythema, erosion, ulceration, luminal narrowing, focal nodular surface, rigidity along the intestinal wall, and the disappeared haustra coli could be detected in our patients. Eight cases only had dark-purple discolorations of the mucosa without other changes (57.1%, 8/14). Colonoscopy revealed multiple erosions and ulcers in 6 patients (42.9%, 6/14). Patient with luminal narrowing in the hepatic flexure was endoscopically observed in one patient (7%, 1/14). The characteristic endoscopic view is presented in Figure 2. Transverse and ascending colon were the most frequent location, the distinctive endoscopic features were also presented in terminal ileum, left hemicolon, and rectum in our cases. Concomitant rectum malignancy (1 case) and colon polyps (2 cases) were found in our group. Biopsy was happened in 5 patients, 3 patients showed chronic inflammation of mucosa, 1 patient presented infiltration with eosinophilia, and 1 case found necrosis of mucosa.

### 3.4 Treatment

**Figure 3:**
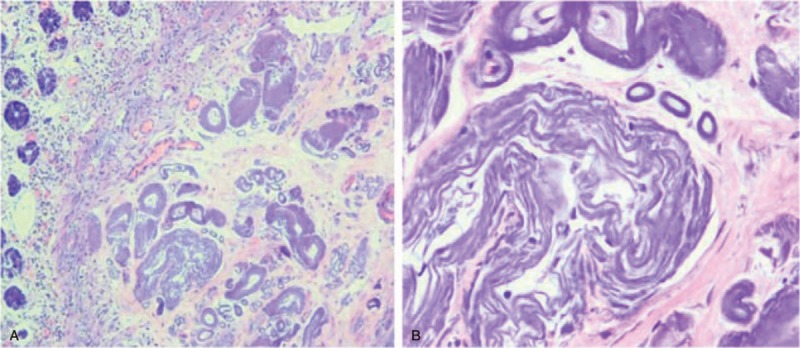
Histologic findings. Microscopic examination showed multiple calcifications in the vascular of marked thickening colon. (A) H&E ×50. (B) H&E ×200.

In our study, only 4 patients (16%, 4/25) had the history about long-term use of Chinese herbs and medical liquor who were asked to discontinue. The conservative treatment with close follow-up was preferred in our group. In 5 cases with no obvious abdominal symptom, only follow-up was indicated with low-residue diet. The conservative management mainly contained bowel rest, improvement of microcirculation, prophylactic anti-infection, other symptomatic, and supportive treatment. Alprostadil injection was the most common therapy used in our center. The medicine of Hirudo extract was also tried in some patients with no tendency of hemorrhage. Nutritional support treatment, probiotics supplementation were also applied. Two patients with repeatedly intestinal obstruction accepted the hemicolectomy. A patient with abdominal pain obtained symptomatic relief following conservative treatment but serious relapse later with perforation in the hepatic flexure eventually resulted in surgery. In 3 surgical patients, the histopathologic findings were similar, indicated the intestinal wall and mesenteric venous fibrosis, hyalinization, calcifcations, ulceration of the colonic mucosa, wall thickening, all of which confirmed the diagnosis of PC (Fig. 3). Through the image of abdomen CT, the extent of calcifications in these 3 surgical patients was also severe (Table 2).

**Table 2:**
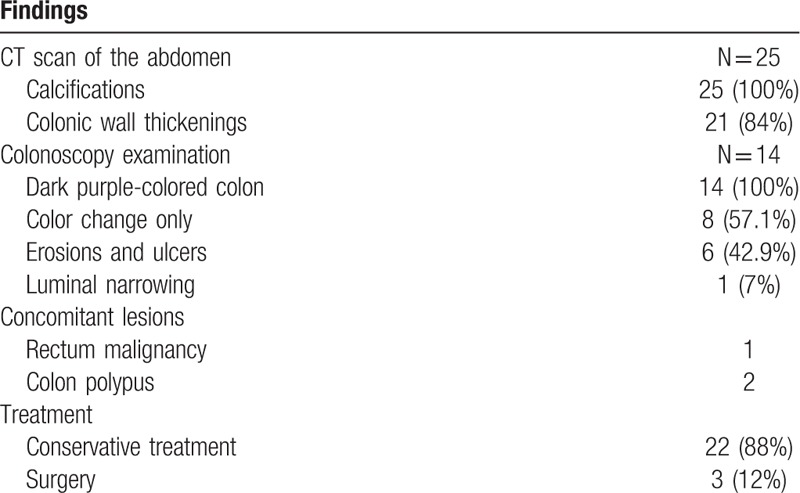
The radiologic/endoscopic findings and treatment.

## 4 Discussion

Koyama et al^[4]^ brought this new disease entity to our attention and named the condition in 1991. In 2000, to distinguish it from common ischemic colitis caused by arterial diseases, Yao et al^[5]^ named this rarity “phlebosclerotic colitis.” In 2003, Iwashita et al^[6]^ proposed the term “idiopathic mesenteric phlebosclerosis” (IMP) because of its nonthrombotic stenosis or occlusion due to marked fibrous thickening of the venous walls with calcifications. PC was a rare, but characteristic entity which should be kept in mind for clinicians, it would initially misdiagnose with other diseases such as inflammatory bowel disease (IBD). Interestingly, PC was almost exclusively observed in the Asian population, mostly from Japan, followed by China and Korea. In Japan, the prevalence in 0.01 case per 100,000 people.^[7]^ Guo et al^[8]^ detailed analyzed the clinical data of all 52 cases identified from PubMed till 2014, the average age of these patients was 59.8 years and 53.8% of which were men. In our group, we noted that the majority of patients were also old patients (mean age of 63.5 years) and male overwhelmingly.

In spite of the unique known clinicopathologic settings, the pathogenesis of IMP had not been well established and the cause remained unknown. PC was initiated by a slow but longstanding and direct hypoxic injury to the venous muscular layer, which lead to gradual mummification and then sclerosis and calcification in this layer.^[1]^ Certain toxins or biochemicals, probably existing in the frequently ingested contents and absorbed to the venous return, may play important role in this damage. Today, more and more literatures reported that certain substances or toxins from ingested Chinese herbs may contribute to PC.^[9,10]^ In Miyazaki et al's^[11]^ report, PC occurred in the married couple who were both regularly taking Chinese herbs suggested the possibility that the Chinese herbs may have contributed to the pathogenesis. In our research, patients with a history of taking herbal medicines comprised 16%, the other 21 cases denied use of any herbal medication. Dialysis, chronic liver disease with portal hypertension, diabetes, vasculitis, increased intraluminal pressure in the right-sided colon were also reported to be involved in the etiology.^[12]^ An association with a region-specific lifestyle or genetic susceptibility had been speculated, as most reported cases were from East Asia. In our 25 cases, 7 patients had the history of digestive malignant tumors which occupied the significant proportion, so the relationship between tumor and PC needed to be further explored. As is well known, water absorption and some digestive activities predominantly occurred in the ascending colon and cecum. Certain toxic agents may be also absorbed by venous return from the proximal colon which may reveal the reason that PC usually involved the right-side colon.

The symptoms, signs, and laboratory examinations of PC were nonspecific. In general, the clinical symptoms depended on the severity of the disease which was attributable to the ischemic change in the colon secondary to the sclerosis of the draining veins, the onset of process was generally gradual. Furthermore, the extent of mesenteric venous calcification was strongly positively associated with the number of episodes of active disease.^[13]^ The main symptoms contained abdominal pain, diarrhea, abdominal distension, gastrointestinal bleeding, etc. Serious complications including intestinal obstruction and perforation may develop during the advanced stage. In addition, partial patients remained asymptomatic. As in our patient, the most common symptoms were abdominal pain (40%) and intestinal obstruction (16%), followed by diarrhea and gastrointestinal bleeding. Three cases (12%) had no obvious symptoms. Kato et al^[14]^ reported that the most common symptoms were chronic and recurrent abdominal pain and diarrhea, 10% of patients remained asymptomatic.

On colonoscopy, the typical and characteristic finding was dark-purple discolorations mucosa in the affected colon. Shibata's report raised dark purple-colored colon: sign of pathic mesenteric phlebosclerosis.^[15]^ This distinctive endoscopic feature was present in all of our patients. The other endoscopic findings included mucosal edema, erythema, erosion, ulceration, rigidity of the colon wall, loss of normal haustra, luminal narrowing, focal nodular surface, etc. Saito et al^[16]^ endoscopically observed a patient with PC for 3 years and considered deep circumferential ulceration on colonoscopy as the aggressive nature of this disease. Except the right side of the colon, characteristic change in terminal ileum, left hemicolon, and rectum were also observed in our study which may also reveal the advanced phase. Histopathologic characteristics included marked thickening of the venous wall with calcifications, fibrosis, collagen fiber perivascular deposits, narrowing of the lumen with no thrombosis, which mainly involved the submucosa of colonic wall. In some patients, mucosal atrophy, ulceration, necrosis, and inflammation were also found. Our endoscopic biopsy revealed no mucosal fibrosis and calcifications because biopsy specimen only had a small amount of tissue that submucosal vessels could not be taken in general. Lee et al^[17]^ also thought mucosal biopsy may not be sufficient for evaluation of submucosal vessels and anticipated that delayed wound healing by venous congestion and biopsy-induced bleeding could be problematic.

Almost all reported cases shared the characteristic imaging features of calcifications alongside the colonic and mesenteric veins tributaries. Calcifications were predominantly seen within SMV distribution of the ascending/transverse colonic wall and all its peripheral tributaries. All our patients showed varying degrees of calcifications, the range of calcification was reported to be related to the symptoms, and our study confirmed this viewpoint through the image of asymptomatic and surgical patients. Multiple fine, tortuous, thread-like, or serpentine calcifications may be shown in abdomen especially in the right hemicolon region through the examination of abdominal X-ray which needed your careful inspection, however most of the results were negative. CT was believed to be the most valuable imaging modality for the diagnosis of PC and follow-up of patients. In addition to the calcifications, edematous thickening of the colonic wall and increased density in the fatty tissue of the surrounding mesentery were its CT features of this entity. Based on the review of the literature and our experience, the linear calcifications were more clearly seen on the mesenteric side and arranged perpendicular to the long axis of the colon.^[18]^ Three-dimensional reconstruction of CTA was thought to be an ideal choice as it was capable to demonstrate the precise location and extent of the calcifications which could prompt further treatment or investigation.^[8]^ In the report of Lee and Seo,^[19]^ fibrosis and calcification occurred in peripheral mesenteric veins first, and then intramural tributaries of mainly the right hemicolon, accompanied by the sclerosis of muscular wall of the colon. Regarding the more proximal portion, IMV and SMV were involved in more advanced phase affecting the entire colon.

Knowledge of its distinctive endoscopic and radiologic appearance would help you to make the clinical diagnosis and biopsy results were insufficient or nondiagnostic sometimes. Due to the lack of definite calcification of the affected veins in the early phase, accurate diagnosis may be even difficult. In this stage, microscopy only revealed submucosal fibrosis of the colonic wall and thickening of venous wall in the mesentery without calcification. Kusanagi et al^[20]^ thought the obstruction of the colonic or mesenteric veins with collaterals visualized on dynamic or contrast enhanced CT or angiography could be the only sign indicating PC in the early stage. Colonic wall thickening accompanied by calcifications may occur in other diseases like mucinous adenocarcinoma, leiomyosarcoma, schistosomiasis japonica, etc., in the literature report.^[12]^ Clinically, the awareness of this rare disease entity was needed because of its characteristic endoscopic and radiologic findings, especially when ischemic colitis was suspected clinically and imaging. Careful observation of venous obstruction and calcification in the mural and mesenteric veins tributaries through the examination of CT or CTA was necessary in these cases.

Nowadays, although more and more patients had been described in the literature, there was no consensus on the treatment. Conservative treatment with close follow-up was preferred in most reported cases. Patients with long-term use of Chinese herbs and medical liquor were asked to discontinue. In terms of treatment, bowel rest, improvement of microcirculation, prophylactic anti-infection, other symptomatic, and supportive treatment were indicated. Alprostadil injection and the medicine of Hirudo extract were the common therapy used in our center with no tendency of hemorrhage. In addition, reported treatments included anticoagulants and amino salicylic acid preparations.^[21,22]^ Surgery had been suggested for patients with severe complications (perforation, intestinal obstruction, hemorrhage, etc.), patients with more extensive venous calcifications, patients in whom the symptoms persisted or recurred after conservative treatment. Hemicolectomy, subtotal, or total colostomy was considered curative therapy with a relatively good prognosis. When determining to have the operation, the resection range of diseased intestine was crucial. You could judge it through the color of the serosa and intestinal wall thickening findings intraoperatively, the range of calcification on abdominal CT and mucosa changes with colonoscopy preoperatively.^[3]^ In accordance with literature, the majority of patients (88%) in our 25 patients accepted the conservative management, only 3 cases had the surgery of colectomy. A patient obtained symptomatic relief resulted in surgery due to the sudden perforation in the follow-up phase with conservative treatment. In Lin's study,^[23]^ evaluation of the severity and extent of IMP based on the total mesenteric venous calcification score, number of involved colonic segments, and the presence bowel loop dilatation on CT may be useful to indicate the outcomes of conservative treatment and need for surgery.

In summary, PC was a very rare, but characteristic entity with unclear etiopathogenesis. Dark purple-colored colon and calcification along the colon and mesenteric venous would help you to make the diagnosis. CT and CTA were thought to be the most valuable imaging modality for the diagnosis and follow-up. However, there was no standardized treatment nowadays, the management ranged from supportive care to surgery. Further investigation and clinical experience was required to explore its pathogenesis and establish optimal treatment plans. Meanwhile, the relationship between tumor and PC needed to be further explored in the future.

## Author contributions

**Writing – original draft:** Wenguo Chen.

**Writing – review & editing:** Wenguo Chen.

**Data curation:** Huatuo Zhu.

**Methodology:** Hongtan Chen.

**Formal analysis:** Guodong Shan.

**Conceptualization:** Guoqiang Xu.

**Supervision:** Fei Dong, Lihua Chen.

**Validation:** Fei Dong, Lihua Chen.
